# A case report of dermatomyositis with the missed diagnosis of non-small cell lung cancer and concurrence of pulmonary tuberculosis

**DOI:** 10.1515/med-2022-0451

**Published:** 2022-03-04

**Authors:** Yuting Yin, Jing Chi, Yang Bai

**Affiliations:** Department of Respiratory and Critical Care Medicine, Chongqing Shapingba District People’s Hospital, Chongqing, 400010, China; Department of Respiratory and Critical Care Medicine, The First Affiliated Hospital of Chongqing Medical University, Yuzhong District, Chongqing, 400010, China; Department of Respiratory and Critical Care Medicine, The First Affiliated Hospital of Chongqing Medical University, No. 1 Youyi Road, Yuzhong District, Chongqing, 400010, China

**Keywords:** dermatomyositis, lung cancer, pulmonary tuberculosis, missed diagnosis

## Abstract

A 42-year-old man with four months of retrosternal pain and two months of skin rashes and proximal muscle weakness was diagnosed with dermatomyositis (DM) based on muscle enzyme analysis and needle electromyography. Chest computed tomography (CT) showed scattered inflammation nodules in both lungs’ upper lobes with negative sputum smear for lung cancer and pulmonary tuberculosis (TB). A good clinical response to oral prednisone was obtained, except for the retrosternal pain in the preceding two months. Urgent CT pulmonary angiography ruled out pulmonary thromboembolism but revealed squamous cell lung cancer with metastases in the sternum and mediastinal lymph nodes. In retrospect, we found osteolytic destruction consistent with sternal metastasis on CT taken at the initial treatment of DM, which was missed by radiologists. Simultaneously, the man was diagnosed with pulmonary TB based on rapid mycobacterial TB detection. This case report indicates the radiologic errors and highlights the importance of a thorough search for underlying lung cancer and pulmonary TB in patients with DM, especially in countries with a high TB burden.

## 1 Introduction

Dermatomyositis (DM) is an idiopathic inflammatory myopathy manifested by progressive symmetric proximal muscle weakness and pathognomonic skin rashes, including periorbital heliotrope discoloration and Gottron’s papules [1]. DM is associated with the likelihood of simultaneous or sequential lung cancer and is involved in developing pulmonary tuberculosis (TB) [2,3,4,5]. The coexistence of lung cancer and pulmonary TB is a challenge for diagnosing and treating both diseases, which might be due to the association and existence of immune disorders in the tumor environment [6]. We present a case of DM with a missed diagnosis of lung cancer due to the interpretation errors on chest computed tomography (CT) and concurrence of pulmonary TB after invasive bronchial washing and rapid mycobacterial TB detection. The patient has given his written informed consent to publish this case report.

## 2 Case report

**Figure 1:**
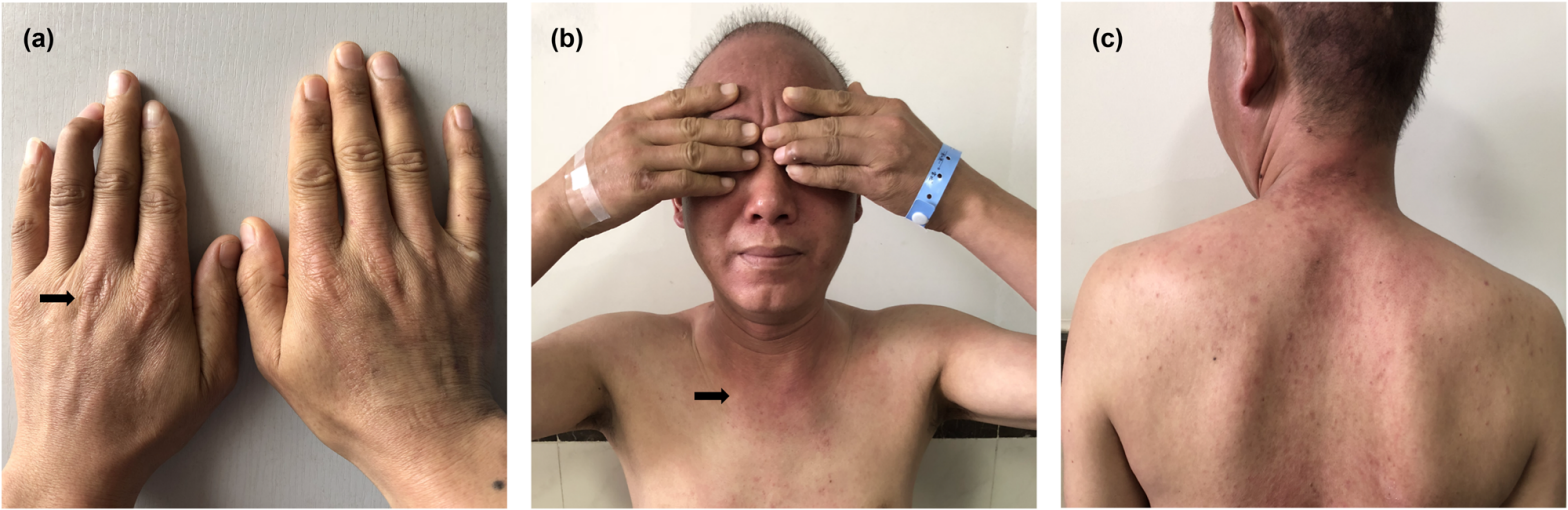
Skin rashes were seen over the dorsal surface of the hands (Gottron’s papules) (a) black arrow, especially over the face and V-neck area (b), black arrow, and the back (c).

**Figure 2:**
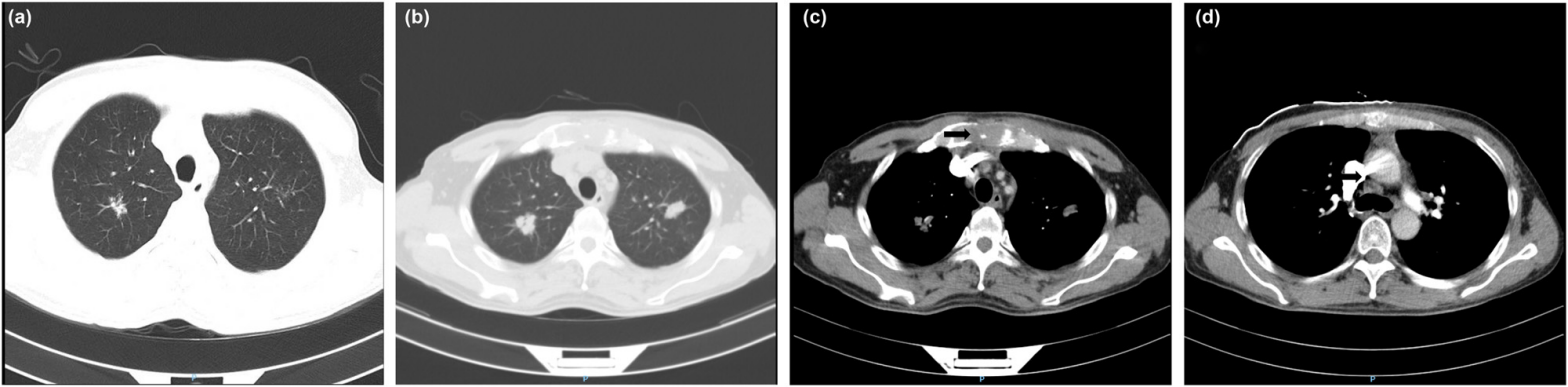
CT scan performed at the initial treatment of DM showing scattered inflammation nodules in lung apices (a), enhanced CT scan performed during this admission showing bilateral apical lung nodules with lobulated and spiculated margins (b), sternal osteolytic destruction (c) black arrow), and mediastinal lymphadenopathy (d) black arrow).

A 42-year-old man with a history of smoking 40 pack-years presented with four months of retrosternal pain and two months of skin rashes and muscle weakness and was referred for muscle enzyme analysis and needle electromyography. The retrosternal pain was described as dull and with mild intensity, worse with deep inspiration and cough. The skin rashes were seen over the dorsal surface of the hands (Figure 1a), especially over the face and V-neck area (Figure 1b), and the back (Figure 1c). The muscle weakness was noticed when climbing stairs, rising from a seated position, and reaching for items above shoulders. Muscle enzyme levels were markedly elevated: creatine kinase was 10,565 U/L (reference <310 U/L), aspartate transferase 509 U/L (reference <46 U/L), and myoglobin 1,622 µg/L (reference <72 µg/L). The lactic dehydrogenase level was also significantly increased to 1,063 U/L (reference <250 U/L). Needle electromyography showed abnormal spontaneous activity and neurogenic recruitment in bilateral biceps and quadriceps. The CT scan of the chest at the initial treatment revealed the scattered inflammation nodules at both lungs’ upper lobes (Figure 2a) with repeatedly negative sputum smear for lung cancer and pulmonary TB. The man was diagnosed with DM and then treated with oral prednisone (80 mg per day for four weeks) [7]. A good clinical response was obtained as the regression of skin rashes, recovery from muscle weakness, and improvement in enzyme analysis. He was referred to our department to further evaluate and manage progressive retrosternal pain in the preceding two months.

On admission, the patient was in severe retrosternal pain with aggravation on deep inspiration. Physical examination showed new sternal tenderness and the same skin rashes over the face, neck, upper trunk; the vital signs were stable. Blood coagulation analysis demonstrated an elevated D-dimer level; the electrocardiogram, oxygenation, and other laboratory tests were normal. Both DM and its associated treatment are risk factors for venous thromboembolism [8]. Based on the presence of chest pain and elevated D-dimer level, urgent CT pulmonary angiography was performed and displayed good contrast filling in the pulmonary vessels without evidence of pulmonary thromboembolism. The contrast-enhanced CT scan showed bilateral nodules with lobulated and spiculated margins (Figure 2b), sternal osteolytic destruction (Figure 2c), and mediastinal lymphadenopathy (Figure 2d). The man was diagnosed with squamous cell lung cancer (T4N3M1c, stage ⅣB) based on positive endobronchial ultrasound-guided transbronchial needle aspiration of node station seven and needle biopsy of the sternum. The patient then received combined chemotherapy with gemcitabine (1,000 mg/m^2^) and nedaplatin (80 mg/m^2^), to which he showed partial response. We analyzed the CT scan taken at the initial treatment of DM and found sternal osteolytic destruction in the mediastinal window setting responsible for the patient’s retrosternal pain. Simultaneously, he was diagnosed with pulmonary TB based on positive rapid mycobacterial TB detection of bronchial washing and then received standard anti-TB therapy composed of isoniazid (300 mg per day), rifampicin (450 mg per day), pyrazinamide (1,200 mg per day), and ethambutol (750 mg per day). The patient died of progressive lung cancer 18 months following the diagnosis.


**Ethics approval and consent to participate:** This study was conducted following the Helsinki Declaration II and was approved by the Institutional Review Boards of The First Affiliated Hospital of Chongqing Medical University. The patients provided written informed consent for publication of individual clinical details and all the accompanying images.

## 3 Discussion

DM is the most common inflammatory myopathy characterized by specific skin rashes and progressive proximal muscle weakness. It is associated with an increased risk of simultaneous or sequential malignancy, which occurs primarily in the ovary, gastrointestinal tract, lung, and breast [9]. The risk of malignancy is highest in the first year after the diagnosis of DM and persistent beyond the fifth year, and malignancy has adverse effects on mortality and healthcare cost [10]. Patients with DM are suggested to evaluate malignancy based on gender, age, and ethnicity and strongly recommended long-term follow-up. In this case report, the patient was diagnosed with metastatic lung cancer after recognizing the sternal destruction on the CT scan, which was initially reported as “scattered inflammation nodules but no destructive lesions” by a consultant radiologist. Errors do still occur in the reporting of radiologic images since Garland had demonstrated in 1959 that reporting error rates in daily practice were 3–5% when negative studies were included, and the rate was 32% when positive studies were retrospectively analyzed [11]. This missed diagnosis could be attributed partially to the first abnormality’s search satisfaction (scattered inflammation nodules) and under-reading of visible abnormality (sternal osteolytic destruction), which are the two major radiologic errors [12]. After identifying the first abnormality, radiologists are prone to stop searching for another abnormality. And the search satisfaction is also due to excess workload, which increases the probability of radiologic errors accompanied by decreasing image analysis time [13]. Various strategies have been proposed to reduce the radiologic errors and the chance of missed or delayed diagnoses. The use of checklists and standardized reports has been thought to reduce search satisfaction and under-reading, but risk focusing on the lists and ignoring unexpected findings [14]. The use of computer-aided detection has been demonstrated to reduce the likelihood of missing subtle lung nodules and exhibit increased sensitivity but decreased specificity [15]. Although the combination of the two methods mentioned above is promising, it has not been adequately validated in the practice of radiology. Communication between radiologists and referring physicians could also reduce radiologic errors and ultimately benefit patients [16]. The information depending on patient-specific characteristics and physician’s perception of specialization is always needed by the referring physicians from the radiologists [17].

The patients with DM are also at greater risk of developing pulmonary and extrapulmonary TB, especially those receiving long-term corticosteroid therapy [3]. High suspicion for TB in patients with DM should be considered in developing countries with high TB burdens, such as India, Indonesia, and China, which accounted for 45% of the estimated incident cases worldwide [18]. Without evidence of osteolytic bone destruction, this case would represent a diagnostic dilemma as apical regions are frequent pulmonary TB locations, and differential diagnosis between early lung cancer and sputum smear-negative pulmonary TB could be complicated. Pre-existing TB is an independent risk factor for lung cancer and vice versa. In this case, we could not tell which came first due to the lack of previous clinical data. The increased lactic dehydrogenase level might be a good marker for lung cancer screening, which did not apply in this case because DM could also cause its increase [19]. The presence of lung cancer might have resulted in missed diagnosis and delayed treatment of pulmonary TB without conducting electronic bronchoscopy and analysis for TB infection. CT-guided transthoracic core-needle biopsy could also be performed in this case and facilitate the differential diagnosis of lung cancer and pulmonary TB or to arrive at a definitive diagnosis of both diseases simultaneously.

## 4 Conclusion

This case report indicates the importance of a thorough search for underlying lung cancer and pulmonary TB in patients with DM, especially in countries with a high TB prevalence. Radiologic similarities often pose a diagnostic dilemma between early lung cancer and sputum smear-negative pulmonary TB. Therefore, multiple invasive and noninvasive investigations should be performed to rule out or confirm the alternative diagnoses.
